# An Inter-Laboratory Validation of a Real Time PCR Assay to Measure Host Excretion of Bacterial Pathogens, Particularly of *Mycobacterium bovis*

**DOI:** 10.1371/journal.pone.0027369

**Published:** 2011-11-14

**Authors:** Emma R. Travis, William H. Gaze, Alessandra Pontiroli, Francis P. Sweeney, David Porter, Sam Mason, Matthew J. C. Keeling, Rebecca M. Jones, Jason Sawyer, Alicia Aranaz, Elena Castellanos Rizaldos, Jennifer Cork, Richard J. Delahay, Gavin J. Wilson, R. Glyn Hewinson, Orin Courtenay, Elizabeth M. H. Wellington

**Affiliations:** 1 University of Warwick, School of Life Sciences, Coventry, United Kingdom; 2 Animal Health and Veterinary Laboratories Agency, Weybridge, United Kingdom; 3 School of Veterinary Sciences. Centro de Vigilancia Sanitaria Veterinaria (VISAVET) and Animal Health Department. Universidad Complutense de Madrid, Madrid, Spain; 4 Food and Environment Research Agency, Sand Hutton, York, United Kingdom; Auburn University, United States of America

## Abstract

Advances in the diagnosis of *Mycobacterium bovis* infection in wildlife hosts may benefit the development of sustainable approaches to the management of bovine tuberculosis in cattle. In the present study, three laboratories from two different countries participated in a validation trial to evaluate the reliability and reproducibility of a real time PCR assay in the detection and quantification of *M. bovis* from environmental samples. The sample panels consisted of negative badger faeces spiked with a dilution series of *M. bovis* BCG Pasteur and of field samples of faeces from badgers of unknown infection status taken from badger latrines in areas with high and low incidence of bovine TB (bTB) in cattle. Samples were tested with a previously optimised methodology. The experimental design involved rigorous testing which highlighted a number of potential pitfalls in the analysis of environmental samples using real time PCR. Despite minor variation between operators and laboratories, the validation study demonstrated good concordance between the three laboratories: on the spiked panels, the test showed high levels of agreement in terms of positive/negative detection, with high specificity (100%) and high sensitivity (97%) at levels of 10^5^ cells g^−1^ and above. Quantitative analysis of the data revealed low variability in recovery of BCG cells between laboratories and operators. On the field samples, the test showed high reproducibility both in terms of positive/negative detection and in the number of cells detected, despite low numbers of samples identified as positive by any laboratory. Use of a parallel PCR inhibition control assay revealed negligible PCR-interfering chemicals co-extracted with the DNA. This is the first example of a multi-laboratory validation of a real time PCR assay for the detection of mycobacteria in environmental samples. Field studies are now required to determine how best to apply the assay for population-level bTB surveillance in wildlife.

## Introduction

*Mycobacterium bovis* is the causative agent of bovine tuberculosis (bTB) which affects cattle and a wide range of other mammals, including humans. *M. bovis* has been shown to persist in the environment for several months to years [1], [2], [3], [4], [5], raising questions about the role of environmental reservoirs in the chronic persistence of bTB in some cattle herds and wildlife populations [2], [6], [7]. Reservoirs of infection have been reported in wildlife populations in parts of the United Kingdom, Republic of Ireland, North America, Africa and New Zealand [8]. In the United Kingdom and the Republic of Ireland the Eurasian badger (*Meles meles*) is implicated in the persistence of *M. bovis* in cattle [9], [10].

In the industrialised world, where zoonotic human tuberculosis incidence is low, the main impact of the disease is economic with losses in agricultural productivity. In the UK, despite an ongoing test and slaughter programme for cattle and periods of statutory badger culling, there has been an average 18% increase in the annual number of new confirmed cattle herd breakdowns since the mid-1980s [11] with an estimated cost of approximately £108 million ($175 million) in 2008–2009 [12]. In parts of the developing world where there are few animal control measures in place, infection in cattle can also have a significant impact on human health [13]. The WHO has recently designated bovine tuberculosis as a neglected zoonosis, with particular reference to the developed world [14]. A further issue for concern is the transmission of *M. bovis* from livestock into wildlife reservoirs in free-ranging ecosystems. *M. bovis* has become established in the African buffalo (*Syncerus caffer*) populations within South Africa's Kruger National Park [15] and been observed in a number of other species, from primates to predators, including lions [16], [17]. White-tailed deer are now considered to be the primary reservoir and maintenance host of bTB in Michigan, USA [18].

*M. bovis* transmission between wildlife, livestock and humans is expected to be primarily via aerosol routes of contact, however there is growing evidence to suggest that the environment may be a potentially important reservoir of the organism [19], [20], [21]. Furthermore, current methods for *M. bovis* detection in wildlife involve invasive trapping and sampling [18], [22], a time-consuming and expensive process. The development of a non-invasive and sensitive tool to detect *M. bovis* in animals and their immediate environment would make a valuable contribution to bTB surveillance and epidemiological studies. Monitoring excretion, rather than infection, is of particular relevance because excreting (i.e. infectious) animals are responsible for transmission. Molecular detection methods have been recently developed for quantification of *M. bovis* by real time PCR in environmental samples [7] and further optimised with particular regard to DNA extraction methodology [23]. An inhibition control assay has also been developed [23]. This study validates this molecular assay through rigorous testing in three independent laboratories aimed to assess concordance, reliability and sensitivity. We collected and tested badger faeces from latrines in areas of high and low bTB incidence in cattle. In addition, we spiked a sub-sample of the faeces collected from a low incidence area with known titres of *M. bovis* BCG. In parallel, an inhibition assay was applied to all samples to assess presence of PCR inhibitors and thus to limit false negative results.

## Materials and Methods

### Participants

The three laboratories that took part in the study were at the School of Life Sciences, University of Warwick, Coventry, UK, the Technology Transfer Unit (TTU) at the Veterinary Laboratories Agency, Weybridge, UK, and the Centro de Vigilancia Sanitaria Veterinaria (VISAVET) at Universidad Complutense de Madrid, Spain. At each of the three laboratories, one operator was responsible for all of the DNA extractions and PCR reactions. Globally, three operators performed the experiments. The three laboratories are termed Laboratory A, B and C and the operators referred to as Operator 1, 2 and 3.

### Study design and sample panel

The sample panel comprised of 24 spiked faecal samples (3×8 dilutions) and 300 field samples taken from 30 badger latrines (15 each from areas of low and high relative incidence of bTB incidence in local cattle herds. All necessary permits were obtained for the described field studies from the FERA (Food and Environment Research Agency) and the Badger Trust. The bTB breakdown incidence was calculated using the VetNet TB in Cattle system data (DEFRA) which provides national data on farm level bTB skin tests. The bTB incidence in a 5 km radius from each latrine as calculated per farm per year of bTB testing (2003 to 2008) was between 29% and 40% in the high incidence area (putative positive) whereas no farms within a 5 km radius of the latrines sampled in the low incidence area (putative negative) suffered a bTB breakdown during this period. For each latrine, 10 samples were taken from individual stool samples found in a varying number of dung pits. Here, a latrine was the sampling unit, considered a collection of dung pits from one badger social group, each pit containing at least one stool sample. The high incidence area, Woodchester Park in Gloucestershire, England is the subject of a detailed ongoing longitudinal monitoring of the badger populations [22] allowing allocation of latrines to social groups according to territorial boundaries delineated by a longitudinal bait-marking study performed as previously described [24]. Due to geographical location at territorial boundaries, it was not possible to allocate four of the latrines from Woodchester Park to a definitive social group. Samples in the low incidence areas (setts in Cambridgeshire and Bedfordshire) were collected from a single latrine.

In addition, each operator was given a further sample panel of 24 spiked faecal samples (3×8 dilutions) to extract and quantify by real time PCR at one of the other two participating laboratories. Operator 1 also completed a third sample panel of 24 spiked faecal samples at the laboratory C participating in the study, being the only one to test the assay in all 3 laboratories.

Each panel was randomised and blinded by an independent operator prior to test sample distribution. A PostgreSQL relational database (PostgreSQL Development Group) with a Microsoft Access user interface was utilised to manage this process. Unblinding occurred after all experimental work was complete and all data had been entered into the database.

### Preparation of spiked samples

Sample preparation was performed at Laboratory A. Badger faecal samples used for spiking experiments were collected from a local badger latrine in a low bTB incidence area (Warwickshire, UK), kept at ambient temperature for transport and confirmed to be PCR negative for *M. bovis* by performing four real time PCR tests (methodology as described below) in triplicate on 9 DNA extractions using the FastDNA® Spin Kit for Soil (MP Biomedicals, Solon, OH, USA).

For the spiked samples, empty tubes were labelled with unique barcodes, randomly selected and filled with 0.1 (± 0.02) grams of faeces and then spiked with 20 µl of each of 7 dilutions of *M. bovis* BCG Pasteur from 5×10^2^ cells g^−1^ to 5×10^8^ cells g^−1^ in 10-fold dilutions; one further tube per dilution series was spiked with sterile water and served as a negative control. Samples were then stored at –20°C before processing.

### Field samples

Field samples were collected from 30 latrines (15 from high bTB incidence areas, 15 from low bTB incidence areas as described above) with ten stool samples taken at each latrine; in 5 instances, where less than 10 stool samples were present at a latrine, repeated sampling from one or few stool samples occurred. Sampling took place in the summer of 2009. Woodchester Park data, was used to determine which of these latrines were from social groups with positive individuals. Tests applied were biological culture, IFNγ and Stat-Pak as detailed previously [25]. Four capture and testing events occurred during 2009 and any social group testing positive by any test at any capture and testing event was considered positive. Samples were stored at ambient temperature for transport. In Laboratory C, each stool sample was then split into subsamples referred to as “replicates”. Each sample panel therefore consisted of one replicate of each stool sample (10 from each of 30 latrines), with 300 samples in total. Field collection and sample splitting was performed by an operator independent of the testing or blinding procedures. Aliquoted samples were stored at −20°C. Samples were shipped to each laboratory on dry ice overnight and stored at −20°C on receipt. The samples to be processed at Laboratory C were stored on dry ice overnight to ensure all samples were treated equally.

### Strains and media

*M. bovis* BCG Pasteur was grown, harvested, filtered and quantified as previously [23].

### DNA extraction and PCR amplification

All protocols and reagents for DNA extraction and PCR amplification were standardised between laboratories. The exception being that two of the laboratories (A and B) used ABI prism 7500 Fast Real Time PCR system (Applied Biosystems Inc, CA, USA) and the third (Lab C) used BioRad iCycler iQ™ (Bio-Rad Laboratories Inc, CA, USA). An equivalence experiment was performed across all three platforms to ensure reproducibility with no significant differences observed (data not shown).

DNA was extracted using the FastDNA® Spin Kit for Soil (MP Biomedicals, Solon, OH, USA) with the following modifications to the manufacturer's instructions: 0.1 g of sample was extracted and a Precellys®24 (Bertin, Montigny-le-Bretonneux, FR) instrument was used instead of the recommended Fastprep® instrument, to ribolyse samples at 5500 cycles per min for 30 sec in the Lysing Matrix tubes provided. Extracted DNA was stored at −20°C for at least 12 hrs before processing.

Real time amplification of the specific RD4 region of *M. bovis* in faecal DNA extracts was performed as previously described [7], with the following modifications: template DNA was diluted 5 fold and 5 µl added to each well. This was to limit pipetting errors with small volumes. In addition the 2×TaqMan environmental PCR Master mix (Applied Biosystems Inc, CA, USA) was used. Genomic equivalent standards were used to generate a standard curve for the real time PCR using genomic DNA obtained from a filtered culture of *M. bovis* BCG over a dilution range from 8.5×10^5^ to 8.5×10^−1^ genome copies per PCR reaction as described previously [23]. DNA standards were run in triplicate on the same plates as the unknown samples. Triplicate no template control wells were included on each plate.

In order to assess potential inhibition by contaminants co-extracting with the DNA, an inhibition control assay was used as previously developed [23]. In brief, the RD4-GFPpCR®2.1 plasmid, containing a green fluorescent protein (GFP) sequence flanked by *M. bovis* RD4 region primer sites was added to DNA samples to quantify PCR inhibition thought to result from residual contaminants. PCR amplification was performed as previously described [7] with the appropriate modifications as described above. The difference in Ct values of the samples compared to no inhibition control (NIC) was calculated and was referred to as Delta Ct (ΔCt).

### Interpretation

Samples were considered positive for *M. bovis* if each triplicate Ct value was above the baseline with the threshold set at 0.15 Delta Rn for Lab A and B and on auto for the instrument at Lab C. Thresholds were set based on optimisation and equivalence experiments between the laboratories. Samples with <3 positive Ct values were rerun and if the number of positive Ct values was <3 on the repeat test then the sample was regarded as a negative. If inhibition of PCR was observed, considered a ΔCt of >1.5, then a replicate sample was re-extracted and tested.

For field samples a latrine was defined as positive if ≥1 replicate sample tested positive (with three positive Ct values, as above). Thus the unit of study was the latrine not the replicate sample.

The percentage of all samples at the specified spike dilution testing positive across operators was taken as analytical sensitivity. Quantitative recovery was determined as the percentage of spiked cells that were detected for each sample.

### Statistical Data Analysis

Differences in quantitative recovery were analysed using the non-parametric Kruskal-Wallis analysis of variance, with more detailed pairwise analyses performed using the Wilcoxon rank sum test with a Bonferroni correction. All statistical analyses were performed using STATA/ICv. 11.1 (StataCorp LP, College Station, TX, U.S.A.).

For the field samples, the likelihood of the observed result (p/n) for each single sample was calculated supposing that the probability of getting a false positive is *p_i_* in lab *i* and the probability of getting a false negative is *q_i_*. The likelihood associated with a single sample is:
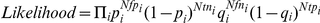
where *Nfp_i_, Ntn_i_, Nfn_i_, Ntp_i_* are respectively the number of false positives, true negatives, false negatives and true positives in lab *i*. The six variables, *p_i_* and *q_i_*, are found with the true distribution of positives across all samples that maximizes the likelihood of the observations.

## Results

### Spiked samples

**Figure 1:**
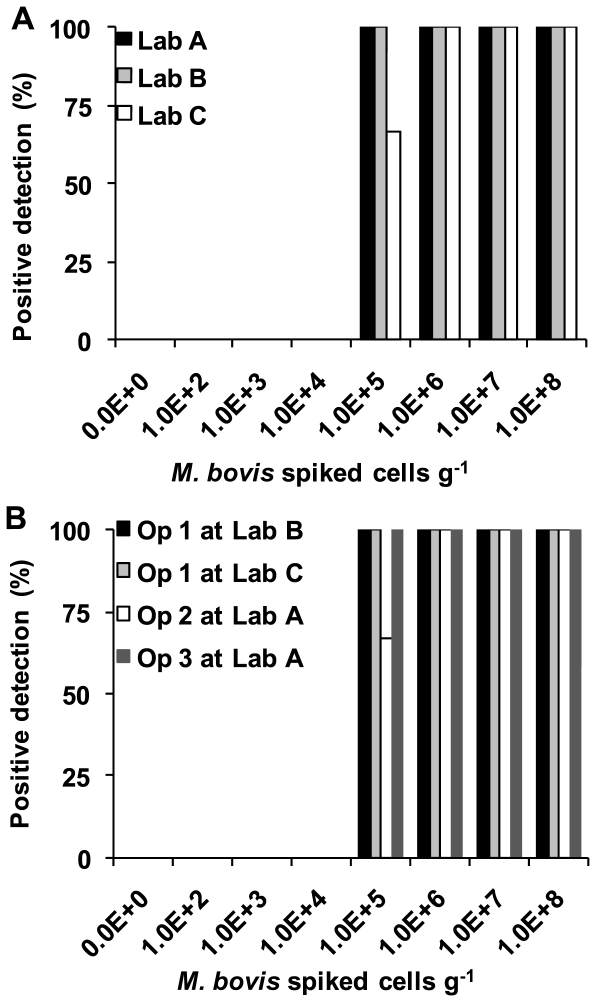
Percentage detection of positive faecal samples spiked with *M. bovis* BCG at a range of cell counts per sample by different operators at different laboratories. A. shows the results from the three operators at their home laboratory. B. shows the results for operators performing the extraction at other than their home laboratory. The keys are shown on the graphs.

**Figure 2:**
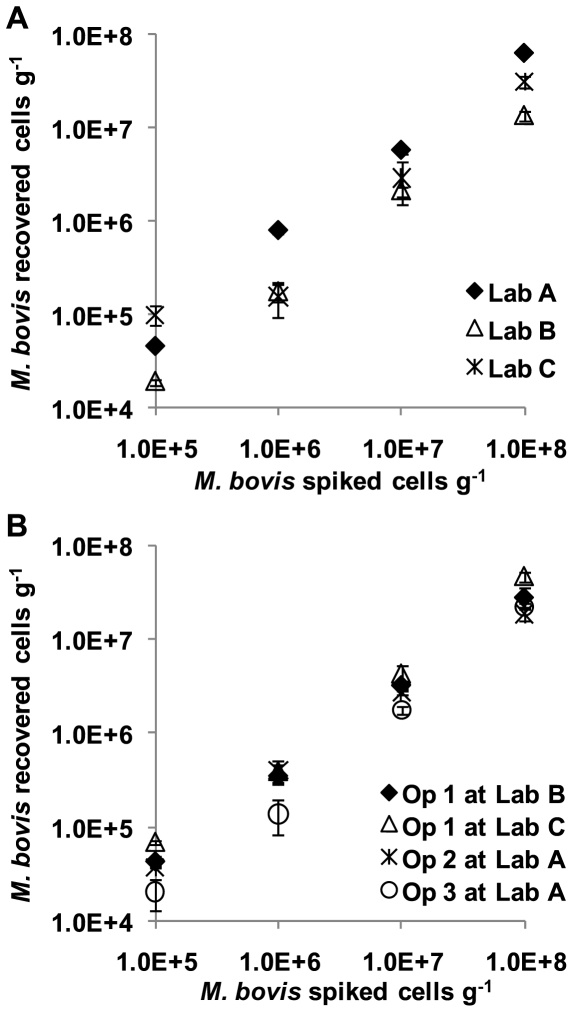
Quantitative cell recovery at the highest four levels of spiking. A. shows the results from the three operators at their home laboratory. B. shows the results for operators performing the extraction at other than their home laboratory. The keys are shown on the graphs.

The panels of spiked samples were tested by an operator at each laboratory. In addition each of the operators tested a further panel at one of the other three laboratories, with one operator assaying a third panel at the third laboratory. In total, therefore, 7 panels were tested. Cell recovery and inhibition were monitored using real time PCR. Figures 1 and 2 show the sensitivity and recovery analysis.

Inhibition was seen to be low with none of the extractions required to be re-extracted based on the inhibition control values: all NICs were within the acceptable range and produced a ΔCt considerably less than 1.5 indicating negligible impurities in the DNA. Real time PCR of the RD4 region showed a clear limit of detection, with no detection at 10^4^ cells g^−1^ and high sensitivity at 10^5^ cells g^−1^ (Figure 1). For the main three panels, performed by each operator at their home laboratory, the specificity of this trial was 100%, with 0% of unspiked samples being detected (0/9) (Figure 1A). No detection of samples with spikes of lower than 10^5^ cells g^−1^ were detected (0/36 0%) whereas at and above 10^5^ cells g^−1^ the sensitivity was 97% with 35 of 36 samples detected.

When all of the seven sample panels were analysed, including those with reciprocal exchanges of personnel, the specificity was 100% with 0% of unspiked samples being detected (0/21) (Figure 1B). No detection of samples with spikes of lower than 10^5^ cells g^−1^ were detected (0/84) whereas at and above 10^5^ cells g^−1^ the sensitivity was 98% (82/84 samples detected).

**Figure 3:**
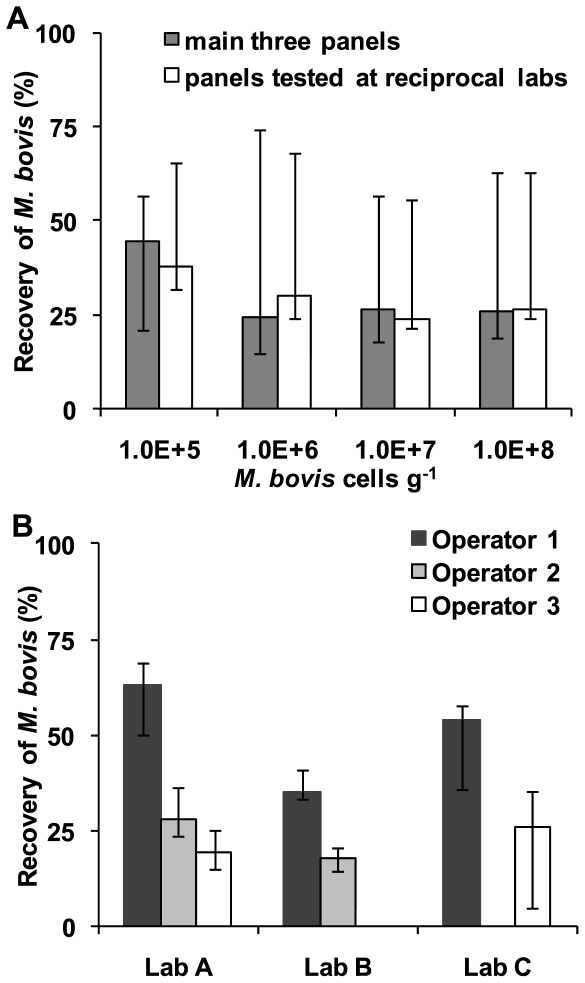
Percentage of spiked cells recovered. A. The three operators at all of the laboratories (N.B. No extractions were performed for Operator 2 at Laboratory C or for Operator 3 at Laboratory B) B. Recovery at the four highest spikes. The keys are shown on the graphs.

The median percentage of absolute cell numbers recovered in each lab varied from 17.7% to 63.3%. The overall median (IQR) recovery for all sample panels was 30.4% (median range: 20.0%–50.5%) (Figure 3). The number of cells per gram did not significantly effect the % recovery of cells over the range of spikes 10^5^–10^8^ cells g^−1^ (p>0.05) (Figure 3A). There were small, but significant (p<0.05) differences between different operators at different labs, between different operators at the same lab and also between the same operator at different labs (p<0.05 with Bonferroni correction) (Figure 3B).

### Field samples

A total of 300 faecal samples collected from badger latrines were tested by each laboratory. As with the spiked samples no inhibition was observed, all NICs were within the acceptable range and a ΔCt of considerably less than 1.5 observed. There was a high degree of concordance between laboratories in the latrines that tested positive, with all three laboratories finding two of the latrines positive that were sampled in the putative positive area. Indeed it was the same subsample from these latrines, taken from the same individual stool sample, that tested positive in all three laboratories. In addition one laboratory (B) also detected a further latrine (2 subsamples) to be positive in the putative positive area, which the other two laboratories did not detect. All samples obtained from the putative negative area tested negative by all three laboratories.

There was also agreement between laboratories with regards to the cells g^−1^ detected in the field samples. Latrine 7 detected positive by all laboratories showed a high cell count of 5.07×10^7^ (Lab A), 1.03×10^8^ (Lab B) and 3.23×10^7^ (Lab C) cells g^−1^. The other latrine detected positive by all three labs (Latrine 14) had a lower cell count, at the lower limit of detection as judged by the spiked panels of 2.04×10^4^ (Lab A), 3.91×10^4^ (Lab B) and 7.51×10^4^ (Lab C) cells g^−1^. The latrine only detected by Laboratory B (Latrine 5), albeit with 2 samples positive, were enumerated at 7.45×10^4^ and 8.60×10^3^ cells g^−1^, again at the limit of detection, as judged by the spiked panels. Given the median % cell recovery of 30.4% (20.0%–50.5%) observed in the spiked panels reported above (Figure 3), the actual cell titres in the field samples were likely to be approximately three fold higher than the counts reported here (i.e. mean cells g^−1^ adjusted for 30.4% recovery; Latrine 7: 2.04×10^8^, Latrine 14: 1.48×10^5^, Latrine 5: 1.37×10^5^)

By comparing the likelihoods from different possible distributions of true positives across the 300 samples, the probability that either of the two samples were in fact false positives but testing positive in all three labs, was p <3×10^−9^. The samples that tested positive in just the one lab are likely to be true positives, although somewhat borderline (p = 0.055). The probability of recording agreement by chance on the samples detected as positive was negligible (p<0.001).

## Discussion

This study establishes that the real time PCR on badger faeces was highly reproducible with high analytical specificity (100%) and sensitivity (97–98%) when trialled between three independent laboratories using spiked samples. The spike panels revealed that the limit of detection for reproducible detection was 1×10^5^ cells g^−1^, with a median (IQR) recovery of 30.4% (20.0%–50.5%). Reciprocal analysis of sample panels by operators performed at a different laboratory to their own also gave good agreement of sensitivity and specificity. Quantitative analysis demonstrated variation between laboratories and between operators within laboratories, however this represented <3-fold differences in absolute (*cf*. log) cell counts which is considered to be small in biological terms. Sensitivity and recovery correlated well with our previous study using the modified FastDNA^®^ Spin Kit on badger faeces [23], where four independent operators within the same laboratory detected *M. bovis* with 75% sensitivity at 4.2×10^5^ with a median (IQR) recovery at the spikes of 4.2×10^5^ and above of 21.5% (13.9%–48.8%). Sensitivity and recovery of *M. bovis* using our PCR assay are comparable with those of other studies using real time PCR to detect pathogenic microorganisms in faeces [23], [26]. The inhibition control assay revealed that there was no contamination of extracted DNA by co-extracting compounds, suggesting a minimal likelihood of false negatives occurring due to chemical contaminants. A previous study had shown that levels of inhibition from DNA extracted from spiked samples by the FastDNA^®^ Spin Kit were low [23] and this study has validated its use for spiked samples as well as more heterogeneous field samples.

Detection in field samples, of unknown status, also showed a high level of concordance between laboratories. None of the 15 putative negative badger latrines tested positive. Of the putative positive latrines (15×10 subsamples) approximately 13% of latrines were recorded by all labs as positive. The same subsamples tested positive in all of the labs. It should be noted that this degree of concordance was high despite *M. bovis* distribution in latrine samples being naturally heterogeneous. This study also revealed that the quantification of *M. bovis* levels in the field samples showed good agreement across all three labs. The probability of achieving these results by chance is negligible.

Whilst this study's primary aim was to assess reproducibility, reliability, sensitivity and specificity, it is of interest to consider possible reasons for the low number of badger latrines testing positive. Previous studies indicated higher prevalence however a different target, the MPB70 antigen gene, was used which is not specific for *M. bovis* and therefore could also have detected other members of the complex such as *M. microti* [21]. Woodchester Park is located in an area of persistently high bTB herd breakdown incidence and the badger faecal samples collected here originated from a single latrine in each of 15 badger social group territories where the resident badgers are routinely monitored for bTB infection using a combination of tests (biological culture, IFNγ and Stat-Pak) [22], [25]. It was possible to match 11 of the latrines to social groups for which test results for 2009 were available, the remaining 4 latrines were not able to be conclusively matched (The Food and Environment Research Agency, unpublished data). Nine of the matched social groups were identified as containing at least one bTB infected animal (testing positive by any test), and of these two social groups showed evidence of excreting *M. bovis* as indicated by culture-positive urine or faeces. All three of the latrines that tested PCR positive in the present study (Latrines 5, 7 and 14) were from social groups which had tested positive with at least one of the tests applied, and of the two social groups observed by culture to be excreting *M. bovis* (Latrines 5 and 9), one was identified by PCR.

The absence of PCR positive latrine samples in the other six matched social groups that tested positive by any means during live capture and test is not surprising, given the number of likely reasons. These include temporal differences in the time of year that the latrine samples were collected and when badgers were captured and sampled; that only a single latrine per social group was tested and by cross-sectional not longitudinal sampling; that excretion of bacilli by infected badgers is intermittent [27], and that cell numbers in latrines may have been below or at the threshold of detection (10^5^ cells g^−1^) by this PCR (e.g. latrines 5 and 14). Importantly, as performed here on latrine samples, the PCR sought to identify excreting animals responsible for transmission, rather than those which may have been infected but currently not excreting.

Whilst this study has focussed on bTB excretion in badgers, the same methodology could be applied for monitoring *M. bovis* in other wildlife species, both those thought to be reservoirs as well as potentially vulnerable species.

To conclude, this ring trial has validated the potential use of this quantitative molecular tool applied to environmental samples, and shown that with spiked samples the test is both reliable and reproducible. With natural samples there was also a high level of concordance between laboratories. This is the first example of a multi-laboratory validation of a real time PCR assay for detection of pathogens in environmental samples. Studies are now required to determine sampling protocols to best apply the assay in the field for purposes of population-level bTB surveillance.
